# Development of Gelatin/Polyvinyl Alcohol Films Incorporated with Blueberry Extracts for Freshness Detection of Shrimp

**DOI:** 10.3390/polym17162188

**Published:** 2025-08-10

**Authors:** Bárbara Teixeira Gomes, Meirielly Jesus, Joana Santos, Clara Suprani Marques, Noé Mitterhofer Eiterer Ponce de Leon da Costa, Fernando Mata, Paulo Cesar Stringheta, Taila Veloso de Oliveira, Nilda de Fatima Ferreira Soares

**Affiliations:** 1 Food Technology Department, Federal University of Viçosa, Viçosa 36570-900, Brazil; barbaratgomes31@gmail.com (B.T.G.); noe.eiterer@mail.uft.edu.br (N.M.E.P.d.L.d.C.); paulocesar@ufv.br (P.C.S.); nfsoares10@gmail.com (N.d.F.F.S.); 2 CISAS—Center for Research and Development in Agrifood Systems and Sustainability, Polytechnic Institute of Viana do Castelo, Rua da Escola Industrial e Comercial Nun’Alvares 34, 4900-347 Viana do Castelo, Portugal; meiriellyjesus@ipvc.pt (M.J.); fernandomata@ipvc.pt (F.M.); 3 Teaching Departament, Federal Institute of Mato Grosso, Campos novo do Parecis 78360-000, Brazil; clara.marques@ifmt.edu.br; 4 Estação Zootécnica Nacional, Instituto Nacional de Investigação Agrária e Veterinária, Av. Vaz Portugal, 2005-048 Vale de Santarém, Portugal

**Keywords:** polymer blend, film characterization, natural blueberry extracts, pH-sensitive, colorimetric indicator

## Abstract

The objective of this study was to evaluate the physical, chemical, mechanical, thermal, and topological properties of polyvinyl alcohol (PVA) and gelatin (GL) films after incorporating three different fractions of blueberry extract: crude extract (EB, without purification), phenolic portion (EF), and concentrated anthocyanins (EA). Additionally, the study aimed to analyze the efficiency of these colorimetric indicator films in monitoring the freshness quality of shrimp. The experiment followed a completely randomized design with one factor—different types of films—studied at six levels: film incorporated with crude blueberry extract (FB), film incorporated with phenolic extract (FF), and film incorporated with anthocyanin extract (FA), in addition to the control films: the plasticized blend containing glycerol, PVA, and GL (FC), the pristine gelatin film (FG), and the pristine PVA film (FPVA). To evaluate the colorimetric sensitivity of the indicators applied to shrimp, storage time was studied at two levels: T0 (before storage—on the day of collection) and T7 (after 7 days of storage at 6.5 ± 1 °C) for the FB and FA films. Regarding thermal properties, the degradation profile occurred in three stages, with the FC film being the most thermally stable. In terms of mechanical behavior, the isolated anthocyanin content increased the elasticity of FA, while the crude extract and other phenolic compounds contributed to the stiffness of FB (Young’s modulus, YM = 22.52) and FF (YM = 37.33). Structurally, the FC film exhibited a smooth and well-blended polymeric surface, whereas FF, FB, and FA displayed heterogeneous and discontinuous phases. The incorporation of blueberry extracts reduced water absorption, leading to decreased swelling and solubility. FF showed the lowest solubility (S = 16.14%), likely due to hydrogen bonding between phenolic compounds and the polymer matrix. Notably, FB demonstrated superior physical, chemical, and mechanical performance, as well as the highest thermal stability among the extract-containing films. It also showed a visible color change (from purple to green/brown) after 7 days of shrimp storage, corresponding with spoilage and pH values unsuitable for consumption. Both FA and FB effectively monitored shrimp freshness, offering a sustainable approach to quality assurance and food waste reduction. Among them, FB was the most practical for visual detection. Overall, these films demonstrated strong potential as pH-sensitive indicators for evaluating the freshness of shrimp.

## 1. Introduction

Blueberries (*Vaccinium* spp.) are fruits rich in phenolic compounds such as stilbens, tannins, and flavonoid compounds, including anthocyanins, flavanones, flavanols, and quercetin [1,2,3], with anthocyanins being the most abundant, followed by phenolic acids [1]. Due to their rich phenolic content, blueberries undergo physicochemical processing to obtain extracts concentrated in these bioactive compounds, which have diverse applications, especially in the food industry. Non-selective extraction using a single organic solvent, such as ethanol, yields a complex extract containing a variety of substances, including sugars, amino acids, anthocyanins, phenolic compounds, flavonoids, and phenolic acids. Once concentrated, this mixture is referred to as crude blueberry extract, characterized by its low purity [1,4,5]. However, by employing purification processes with selective solvents, it is possible to fractionate this crude into two more purified forms: an anthocyanin-rich extract, with higher anthocyanin concentrations; and the phenolic-rich extract, enriched in other phenolics such as phenolic acids, flavanols, and flavan-3-ols in higher concentrations [3,4,5,6].

Some of these phenolic compounds are used as natural pigments and preservatives in various foods, such as sweets and snacks, appealing to health-conscious consumers. Currently, these substances have also gained attention as components in polymeric films due to their antioxidant properties and pH-sensitive indicator dyes. When incorporated into polymer matrices, these compounds can impart intelligent properties, helping to extend shelf life and enable quality monitoring during food storage [1,7,8].

In this context, various biopolymers have been explored to develop sustainable films with functional properties. Gelatin (GL), a thermally processable protein, is frequently used in film manufacturing due to its low cost, biodegradability, and non-toxicity [9,10,11]. Blending GL with polyvinyl alcohol (PVA) is a promising strategy for developing biodegradable films with enhanced characteristics. Moreover, GL is particularly suitable for binding phenolics due to its high content of proline and hydroxyproline residues, which have an affinity for polyphenols such as the anthocyanins in blueberry extract [4,12]. PVA, a biodegradable synthetic polymer, is widely used in combination with natural carbohydrates and proteins to produce blends with improved mechanical properties [7,9,10].

A previous study by Gomes et al. (2024) [4] developed PVA:GL films incorporated with blueberry extracts and demonstrated their ability to change color in response to pH variations, indicating their potential as intelligent food packaging. However, further characterization is necessary for them to be effectively applied as food packaging. It is essential to understand the mechanical and thermal properties of the films, as well as their behavior in the presence of water or humidity, considering that water is a major component of countless food products.

Therefore, the main objective of this study is to expand upon previous work by thoroughly characterizing PVA:GL films containing blueberry extracts and to determine whether, beyond imparting intelligent properties, this bioactive compound enhances their physicochemical performance.

## 2. Materials and Methods

### 2.1. Materials

Blueberry fruits (*Vaccinium myrtillus*) were sourced from the fruit-producing company Quali Fresh (Barbacena, Minas Gerais, Brazil) and stored at −18 ± 2 °C until further use. The polymers used for film preparation were PVA (Sigma Aldrich Co., St. Louis, MO, USA), with a degree of hydrolysis >99% and molecular weight (Mw) between 85,000 and 124,000 g/mol, and a colorless, flavorless, food-grade GL from the Dr. Oetker brand (Lot No. L346036A 22N). Fresh, vacuum-packed, headless gray shrimp (class A) were purchased refrigerated from a local supermarket in Viçosa, Minas Gerais, Brazil.

All chemical reagents used were of analytical grade. Glycerol (GLY) and ethyl acetate were obtained from Vetec Química Fina Ltd.a (Recife, PE, Brazil), while ethanol and methanol were acquired from Merck (Taufkirchen, Bavaria, Germany). Quantitative filter paper (Unifil, Ø 11) was obtained from Dinâmica (São Paulo, SP, Brazil).

### 2.2. Methods

#### 2.2.1. Preparation of Aqueous Concentrate

The extraction of phenolic compounds was carried out according to the methodology described by Gomes et al. (2024) [4]. Briefly, blueberries were crushed using in a mixer (RI1602, Philips Walita, Varginha, MG, Brazil) and combined with 70% ethanol (*v*/*v*) in a 1:10 (*w*/*v*) ratio. The mixture was acidified to pH 2.0 with hydrochloric acid (HCl, 37% *v*/*v*) and subjected to sonication in an ultrasonic bath (Elmasonic TI-H10, Elma, Singen, BW, Germany) at 45 kHz (40 ± 2 °C, 50 min). The resulting mixture was vacuum-filtered (Whatman No. 1 filter paper, Sigma-Aldrich, Saint Louis, MO, USA) and concentrated in a rotary evaporator (RV 10 digital V, IKA, Staufen, Germany) at 100 rpm, 40 ± 2 °C, until complete solvent evaporation. The extraction process yielded, on average, 30% crude extract after evaporation. When necessary, distilled water was added to the concentrated extract to restore the final volume and maintain the same yield profile.

#### 2.2.2. Purification of Aqueous Concentrated Blueberry Extracts

The purification process was performed using a C18 solid-phase extraction cartridge (Sep-Pak Vac 35 cc, Waters, Milford, CT, USA) under vacuum, according to the methodology described by Gomes et al. (2024) [4]. First, the cartridge was conditioned with 50 mL of acidified methanol (0.01% HCl) and 50 mL of acidified distilled water (0.01% HCl). Next, a 50 mL aliquot of the concentrated crude blueberry extract was loaded into the cartridge. The removal of other compounds (such as sugars) was performed with 50 mL of acidified distilled water (0.01% HCl). To obtain the phenolic-rich extract, an additional 50 mL of ethyl acetate was passed through, eluting phenolic compounds while anthocyanins remained adsorbed on the cartridge. To elute the anthocyanins and obtain the purified anthocyanin extract, 50 mL of methanol was passed through the cartridge [5,13]. Following purification, each solvent fraction was evaporated under reduced pressure. The phenolic and anthocyanin fractions that adhered to the inner walls of the collection beaker were reconstituted with 50 mL of distilled water.

#### 2.2.3. Quantification of Phenolic Compounds

This step was performed according to the Folin–Ciocalteau spectrophotometric method Singleton & Rossi Jr (1965) [14]. In test tubes, 0.6 mL of each sample was added separately, with subsequent incorporation of 3 mL of Folin–Ciocalteau reagent and 2.4 mL of Na_2_CO_3_ 7.5%. The mixture was stored in the dark for 1 h. Absorbance was measured at 760 nm on a UV–Vis spectrophotometer (UV-M51, Bel Photonics, Monza, Italy). Quantification was performed using a gallic acid standard curve and the results were expressed as mg of gallic acid equivalent (GAE) per 100 g of blueberry.

#### 2.2.4. Quantification of Total Anthocyanins

It was carried out by the differential pH method, according to Fuleki and Francis (1968) [15]. Absorbance was measured in a spectrophotometer, at pH 1.0 and pH 4.5, at wavelengths of 520 nm and 700 nm. The results were expressed as mg of cyanidin-3-glucoside equivalent (cy-3-glu) per 100 g of blueberry (mg cy-3-glu/100 g).

#### 2.2.5. Production of Colorimetric Indicators (Polymer Blend Based on PVA and GL with Blueberry Extract)

**Figure 1:**
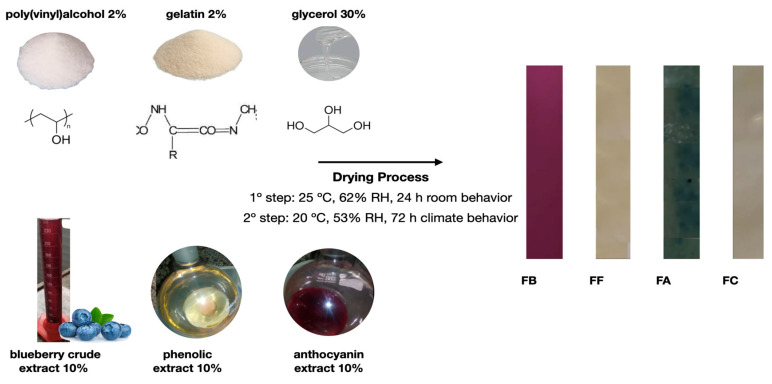
Production of polymeric blends.

The blend films (PVA:GL) were produced by mixing two suspensions: suspension A, containing 2% (*w*/*v*) gelatin in water at 60 ± 2 °C, and suspension B, containing 2% (*w*/*v*) PVA in distilled water at 80 ± 2 °C (suspension B) [4]. Each suspension was stirred for 5 h, then mixed and kept under stirring (300 rpm) until cooled to 40 ± 2 °C (Figure 1). The GL control film was produced with 4% GL and dispersed as suspension A. The PVA control film was produced with 4% PVA and dispersed as suspension B. All film formulations were plasticized with glycerol (GLY) at 30% wt. based on total polymer mass. Blueberry extracts: crude, anthocyanin, and phenolic were incorporated at 10% wt., based on total dispersion volume. The resulting mixtures were homogenized for 20 min at 25 ± 2 °C using magnetic stirring (300 rpm). Subsequently, the suspensions were placed in an ultrasonic bath (40 kHz) for 10 min to remove air bubbles, then poured onto glass plates (33 cm × 9 cm) and dried in two stages: first at 25 ± 5 °C and 62 ± 5% relative humidity (RH), for 24 h, followed by 72 h at 20 ± 2 °C and 53 ± 5% RH in a controlled climatic chamber (420-CLDTS 300, Ethik Technology, Vargem Grande Paulista, SP, Brazil). After drying, the films were vacuum-sealed (200S, Selovac, São Paulo, SP, Brazil) in polyethylene/nylon pouches, wrapped in aluminum foil, and stored at 20 ± 2 °C for further analysis (Figure 1). In total, six films were obtained: (i) FB film, incorporated with crude blueberry extract; (ii) FF film, incorporated with phenolic blueberry extract; (iii) FA film, incorporated with blueberry anthocyanin extract; (iv) FC film, film produced with glycerol (GLY), PVA and GL; (v) FG film, film produced with GLY and GL; and (vi) FPVA film, film produced with GLY and PVA. This follows Gomes et al. [4] with modifications to ensure the final color was clearly visible to the naked eye.

#### 2.2.6. Experimental Design

The experiment followed a completely randomized design. One experimental factor was studied: films produced with different types of blueberry extracts, at six levels (FB, FA, FF) and three controls (FC, FG, FPVA). To evaluate the colorimetric sensitivity of the indicators when applied to shrimp, a separate analysis was conducted for each film (FA and FB), considering the storage time at two levels (T0: before storage—on the day of collection; T7: after 7 days of storage at 6.5 ± 1 °C).

#### 2.2.7. Microstructural Characteristics

The surface morphology of the films was characterized using scanning electron microscope (SEM) images obtained with a low vacuum tabletop microscope (Hitachi Hi-Tech, model TM3000, Tokyo, Japan). Film samples (0.2 × 0.5 cm^2^) were affixed onto stubs using tweezers and a conductive double-sided carbon tape. The electron-accelerating voltage was set to automatic mode. Images were captured at 400× magnification, one image for each polymer film. Subsequently, energy dispersive spectroscopy (EDS) coupled with SEM was carried out to identify the chemical elements present on the surface of each film [16].

#### 2.2.8. Thermogravimetric Analysis

Thermal stability was assessed using a thermogravimetric analyzer (model DTG-60H, Shimadzu, Kyoto, Japan). Approximately 3 mg of each film was heated from 20 °C to 700 °C at a heating rate of 10 °C/min under a nitrogen atmosphere (50 mL/min). Thermogravimetric curves were generated for each polymer film and used for descriptive evaluation of thermal degradation.

#### 2.2.9. Thickness and Mechanical Properties

Film thickness was measured using a digital micrometer (Model 547-401, Mitutoyo, Kawasaki-shi, Japan, with an accuracy of 0.001 mm). Ten random points were measured per sample, and the average thickness was calculated.

Tensile strength (TS, MPa), elongation at break (EB, mm), and Young’s modulus (YM, iMPa) were determined using a universal testing machine (model 3367, Instron Corporation, Norwood, MA, USA) in accordance with ASTM D882 [17]. Film samples (10 × 2.5 cm^2^) were clamped between two grips and stretched at a crosshead speed of 500 mm/min. Three replicates were performed for each treatment.

#### 2.2.10. Grammage

Grammage (G) was determined as the mass (g) per unit area of material (m^2^). For this, square sections of each film (0.1 × 0.1 m^2^) were weighed on an analytical balance. The grammage was then calculated using Equation (1). All treatments were analyzed in triplicate.

(1) $$G\left(g/m^{2}\right)=\frac{mass\left(g\right)}{area\left(m^{2}\right)}$$

#### 2.2.11. Moisture, Swelling Index, and Solubility

To determine moisture content (M, %), film samples (1 × 4 cm^2^) were first weighed to obtain the initial mass (*m*_0_) using an analytical balance (AUY220, Shimadzu, Japan). The film samples were then dried at 105 ± 2 °C for 24 h in an air-circulating oven (400-6ND, Ethik Technology, Brazil) and the dry mass (*m*_1_) recorded. Moisture content was calculated using Equation (2) [18], each treatment was evaluated in triplicate.

(2) $$M\left(\%\right)=(\left(m_{0}-m_{1})/m_{0}\right)*100$$

To assess the swelling index (I, %), the dried film (*m*_1_) was immersed in distilled water at room temperature (25 ± 1 °C) for 24 h. After gently blotting with paper to remove surface moisture, the swollen mass (*m*_2_) was measured. Swelling index (I-%) was calculated using Equation (3) [18], each treatment was evaluated in triplicate.

(3) $$I\left(\%\right)=(\left(m_{2}-m_{1})/m_{1}\right)*100$$

Solubility (S, %) of the films was determined by immersing dried film (*m*_1_) in 50 mL of distilled water (25 ± 2 °C) under constant stirring for 24 h. Followed by drying the samples again at 105 ± 2 °C for 24 h (*m*_3_) [18]. Each treatment was evaluated in triplicate. Solubility was calculated according to Equation (4).

(4) $$S\left(\%\right)=(\left(m_{1}-m_{3})/m_{3}\right)*100$$

#### 2.2.12. Application and Evaluation of the Colorimetric Sensitivity of Indicators in Shrimp

To evaluate the colorimetric sensitivity of the indicator films (FA and FB) in detecting changes in shrimp freshness over seven days of storage, 70 g of shrimps were individually placed into a polypropylene container, in triplicate [19]. A section of the indicator films (2.0 × 1.0 cm^2^) was affixed to the inner surface of the container lid using adhesive tape, exposing a large area of the film to the headspace atmosphere [20]. Then, the containers were wrapped in PVC film and sealed in polyethylene nylon. The systems were stored at refrigeration temperature (6.5 ± 2.0 °C; RH = 66 ± 2.0%) in a glass-lidded display refrigerator, which was exposed to artificial light for seven days. Colorimetric changes in the films (FA and FB) were assessed in triplicate using a colorimeter at 2 points—before storage (L0*, a0**, b0*, h°, C*, and ΔE) and after seven days of storage (L*, a*, b*, h°, C*, and ∆E) [7,20].

#### 2.2.13. pH

Shrimp pH was determined in triplicate according to Method 017/IV of the Institute Adolfo Lutz [21]. For the analysis, 10 g of sample was crushed and homogenized in 100 mL of distilled water [22]. The pH of the shrimp was measured directly before and after seven days of storage using a pH meter (PG 1800, Gehaka, São Paulo, SP, Brazil), previously calibrated with pH 7.0 and 10.0 buffers, at room temperature (25 ± 2 °C).

#### 2.2.14. Monitoring Shrimp Degradation

Shrimp degradation during storage was monitored by evaluating pH (as described in Section 2.2.11) and by assessing the sensory attributes of odor and color. The analyses were performed in triplicate at two time points: T0, on the day of purchase at the local market, and T7, after seven days of storage.

Colorimetric properties of the shrimp were determined using colorimeter (Color Quest XE, HunterLab, Reston, VA, USA) before (L0*, a0*, b0*, h°, C*, and ΔE) and after seven days of storage (L*, a*, b*, h°, C*, and ∆E), in triplicate [6,20]. C*, h°, and ΔE were calculated according to Equations (5)–(7), respectively. The standard conditions employed were D65 illuminant, 10° observation angle, and both CIELAB (L*, a*, b*) and CIELCh (L*, C*, h°) systems. The color coordinates [L* (lightness, where 0 = black and 100 = white), a* (green [–] to red [+]), and b* (blue [–] to yellow [+])] were determined. Using these values, the hue angle (h°, where 0°/360° = red, 90° = yellow, 180° = green, and 270° = blue) was calculated according to Equation (5), the Chroma (C*, which represents the color intensity or saturation ranging from gray to pure color) according to Equation (6) [23], and the overall color difference (ΔE) according to Equation (7) [24].

(5) $$h{}^{\circ}=\arctan(b^{*}/a^{*})$$

(6) $$C^{*}=\sqrt{{a^{*}}^{2}+{b^{*}}^{2}}$$

(7) $$\Delta E=\sqrt{{\left(∆L\right)}^{2}+{\left(∆a\right)}^{2}+(∆{b)}^{2}}$$

In which ΔL represents the variation in the L* coordinate (ΔL* = L* − L0*), Δa the variation in the a* coordinate (Δa = a* − a0*), and Δb the variation in the b* coordinate (Δb = b* − b0*).

#### 2.2.15. Statistical Analysis

Data were analyzed using analysis of variance (ANOVA), and significant differences between means were determined by Tukey’s post hoc test. Assumptions for ANOVA were verified using a Kolmogorov–Smirnov (normality of residuals) and Bartlett’s test (homogeneity of variances). A significance level of *p* < 0.05 was adopted for all analyses. Statistical procedures were conducted using Statistica^®^ software version 7.0 (Statsoft Inc., Tulsa, OK, USA). All results were expressed as mean ± standard deviation.

## 3. Results and Discussion

### 3.1. Bioactive Compounds from Blueberry Extracts

The content of phenolic compounds and anthocyanins determined for crude, anthocyanin, and phenolic blueberry extracts. The crude extract obtained had a total phenolic content of 1392 mg GAE/100 g blueberries and a total anthocyanin content of 252 mg cy-3-glu/100 g blueberries. The purified anthocyanin extract contained a total phenolic content of 901 mg GAE/100 g blueberries and a total anthocyanin content of 244 mg Cy-3-glu/100 g blueberries. The phenolic extract, in turn, presented a lower phenolic content than the extracts mentioned above, presenting a total of 285 mg GAE/100 g of blueberries and no detectable anthocyanin content of 0 mg Cy-3-glu/100 g of blueberries. This can be attributed to the prevalence of anthocyanins as the primary phenolic compounds in blueberries, followed by phenolic acids [1,4].

### 3.2. Scanning Electron Microscopy

**Figure 2:**
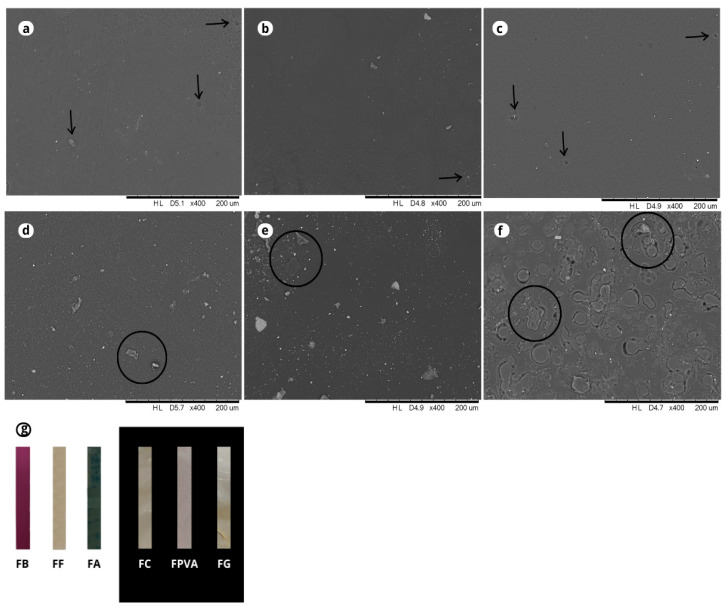
SEM micrographs depict the surface micrography of PVA and gelatin-based films incorporated with different types of blueberry extracts. (**a**) FC; (**b**) FPVA; (**c**) FG; (**d**) FB; (**e**) FF; (**f**) FA; and (**g**) Photography of the films. FC: film produced with PVA and gelatin; FPVA: film produced with PVA; FG: film produced with gelatin; FB: film produced with the crude extract; FF: film produced with the phenolic extract; and FA: film produced with the anthocyanin extract. Micropores or non-dispersed polymers are indicated by black arrows and spots are highlighted with black circles.

The surface morphology of the films was characterized by scanning electron microscopy (SEM), as shown in Figure 2.

The control films exhibited homogeneous, continuous, and smooth surfaces, according to the images of the SEM (Figure 2a–c), suggesting compatibility between the GL and PVA polymers. These observations are consistent with previous studies by Rashid et al. [25] and Tymczewska et al. [26], which reported well-integrated, compact, and non-porous or small-homogeneous pores (<1.5 µm in diameter) in PVA-GL films. Furthermore, macroscopic observation confirmed that the control films were colorless, transparent, and free of phase separation, consistent with the polymer blend literature (Figure 2g) [27].

SEM images also revealed the episodic presence of micropores and non-dispersed polymer in films, including those without extracts, likely resulting from the glycerol volatilization (30%) or the casting procedure (indicated by the black arrows in Figure 2) [22]. These pores are commonly attributed to the entrapment of air bubbles during casting, as similarly reported by Zeng et al. [10] for mulberry anthocyanin-containing films. Despite the presence of micropores, no cracks or fractures were observed in any formulation, supporting the high flexibility and cohesion provided by the PVA-GL-GLY system, as corroborated by Rashid et al. [25]. On the other hand, in the study by Oyeka et al. [28], the GL and PVA film exhibited increased surface heterogeneity with discontinuous cracks and the presence of discrete white spots and agglomerates, which may have resulted from inadequate homogenization during film preparation. These authors achieved better interaction and a more cohesive structure in the film reinforced with 5 wt% cellulose nanocrystals.

Moreover, the PVA and GL films with blueberry extracts (Figure 2d–f) exhibited greater surface heterogeneity than the control films (Figure 2a–c). The appearance of discrete white spots and agglomerates may be attributed to the presence of non-dispersed polymer [29]. Specifically, FA, containing the blueberry anthocyanin-rich extract, exhibited the most heterogeneous surface among the extract-containing films (FF and FB), with visible micro-agglomerates and irregularities. This effect can be explained by the lower number of free hydroxyl groups in anthocyanins compared to other phenolics, such as phenolic acids and flavonoids, limiting hydrogen bonding with the PVA–GL matrix. Furthermore, the predominantly cationic nature of anthocyanins, along with their differing electronegativity relative to the polymer components (PVA and GL), may have further hindered molecular interaction and phase compatibility. Regarding the energy dispersive X-ray spectroscopy analysis (EDS spectrum), the main chemical elements present on the surface of the films were carbon, oxygen, and nitrogen, which are consistent with the molecular composition of PVA, GL, GLY, and the phenolic compounds of blueberry extracts [25,28].

### 3.3. Thermogravimetric Analyses

**Figure 3:**
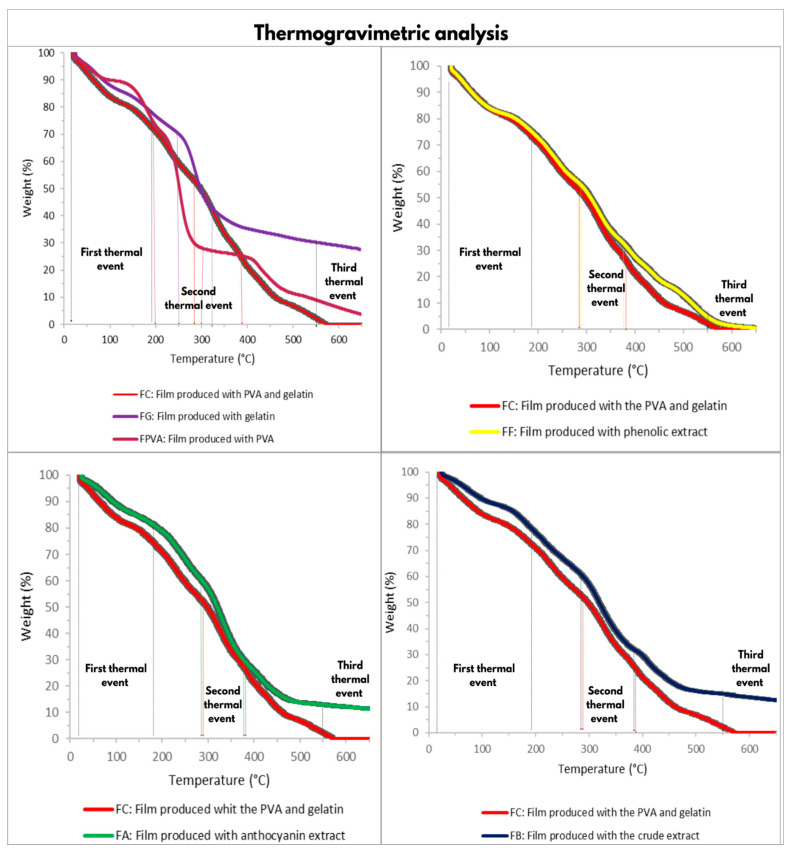
Thermogravimetric curves of the films, delineating the different thermal degradation events in vertical dotted lines.

**Figure 4:**
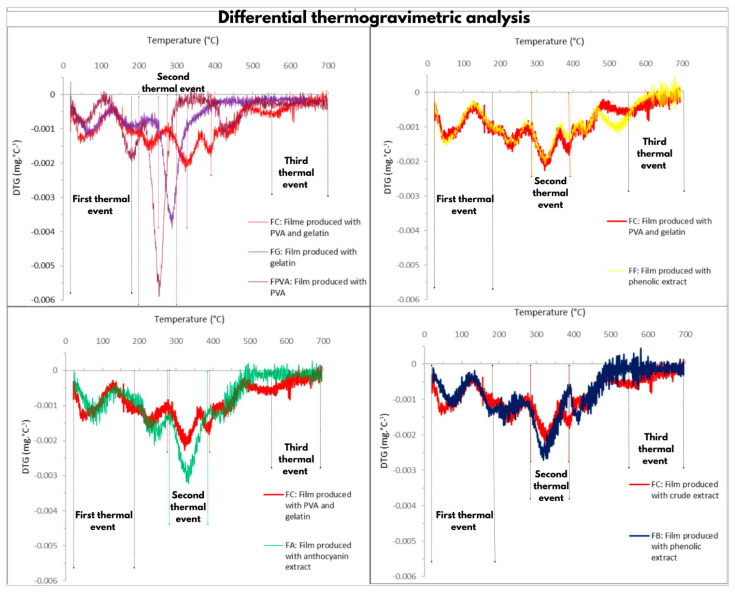
Thermogravimetric derivative of the films, delineating the different thermal degradation events in vertical dotted lines.

The thermal properties of the films, with and without blueberry extracts, were assessed using the thermogravimetric (TG) and derivative thermogravimetric (DTG) analyses, as shown in Figure 3 and Figure 4, respectively. The DTG curves (Figure 4) reveal that thermal degradation occurred in three distinct stages. For all formulations, the first thermal degradation event occurred between 20 °C and 180 °C, corresponding to approximately 20% mass loss (Figure 3). This stage is mainly related to the evaporation of water and the volatilization of the GLY plasticizer present in the polymer matrix [30,31]. In the films containing blueberry extracts, this stage also included the loss of low molecular weight and volatile compounds originating from the extracts [22].

The PVA control film exhibited a second event between 200 °C and 300 °C, with a 70% mass loss and a maximum degradation peak at 270 °C (Figure 3). This stage can be attributed to the decomposition of PVA [31]. In contrast, the FGL exhibited a second event at temperatures between 250 °C and 320 °C, with a 50% mass loss and a maximum degradation peak at 298 °C, attributed to the decomposition of gelatin [31]. The second thermal event of the blend films (FC) and the extract-containing films (FB, FA, and FF) occurred at higher temperatures, between 290 °C and 370 °C, with a 60% mass loss of 60% (Figure 3). This increase in thermal stability is likely due to the combined decomposition of PVA, GL, GLY, and the extracts [32].

The differences in mass loss between the films containing blueberry extracts and the control samples may be related to higher residual water content in the extract-based films [30]. This moisture is likely associated with interactions between water molecules and functional groups present in extra constituents, such as sugars, acids, phenolic compounds, and anthocyanins [30]. Indeed, FB, FA, and FF exhibited higher moisture content than the control films FC, FPVA, and FG.

The third thermal event occurred similarly for all treatments at temperatures above 550 °C. This thermal degradation is due to degradation and carbonization reactions of organic materials within the films [31,33,34].

In the DTG graph (Figure 4), all decomposition peaks of the polymer blend films slightly shifted to higher temperatures when compared to the pristine films, containing only PVA or gelatin. Therefore, it can be concluded that the PVA-GL polymer exhibits superior thermal stability, suggesting that its application may be more advantageous for food packaging purposes than films based on individual polymers.

The addition of the crude extract and the blueberry anthocyanin extract enhanced the thermal stability of the PVA and GL film, while the addition of the phenolic extract did not alter the thermal stability of the polymer blend. In FB and FA, an increase in the maximum degradation peak observed as the concentration of phenolic compounds in the film increased, indicated improved thermal stability (Figure 4). This result may be related to the fact that phenolic compounds act as effective thermal stabilizers due to their radical scavenging properties [35,36]. FB and FA were formulated with the crude extract and the blueberry anthocyanin extract, respectively, both of which are rich in antioxidant phenolic compounds [4]. These findings suggest that the presence of antioxidant-rich blueberry extracts positively influenced the thermal properties of the PVA and GL polymer blend.

### 3.4. Thicknesses and Mechanical Properties

**Figure 5:**
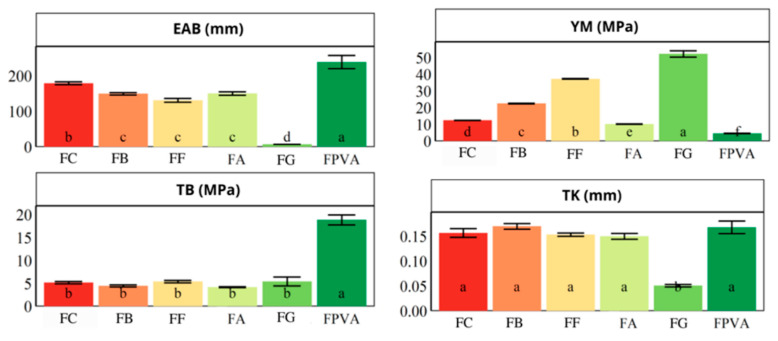
Mechanical properties and thickness of the films. FC (film produced with the PVA and gelatin), FPVA (film produced with the PVA), FG (film produced with the gelatin), FB (film produced with the crude extract), FF (film produced with the phenolic extract), and FA (film produced with the anthocyanin extract). Means followed by the same letter, in the bars, do not differ statistically using the Scott Knott test at a significance level of 5%. TK: thickness; TB: tensile strength at break; EAB: elongation at break; YM: Young’s modulus. All measurements were carried out in triplicate.

The thickness (TK) and mechanical properties of the films were determined and are shown in Figure 5.

The thickness of the investigated films ranged from 50 to 170 μm. In comparison, the thickness (TK) of the GL and PVA films evaluated by Haghighi et al. [37] ranged from 42 to 48 μm, which is lower than those observed in the present study, likely due to methodological differences and component concentrations. Haghighi et al. [37] prepared films with GL and PVA (3% by weight) in a 1:1 ratio, with 25 g of glycerol per 100 g of dry polymer, and bacterial cellulose nano-whiskers were also added. The films were obtained by casting 20 mL into 14.4 cm diameter Petri dishes.

As shown in Figure 5, the incorporation of blueberry extracts did not result in significant changes in film thickness (*p* ≥ 0.05) compared to the control films (FC and FPVA). Furthermore, the GL film (FG) displayed the lowest thickness (0.05 ± 0.01 mm) (*p* < 0.05). In general, polymer blends are associated with more uniform thickness distributions [25]. Similarly, Jebel et al. [20] reported that the addition of eggplant skin extract did not affect the thickness of GL films.

Regarding mechanical properties, the Young’s modulus (YM) of the blend film was intermediate. Pristine GL films had the highest rigidity (*p* < 0.05), whereas the PVA film was the lowest rigidity and most flexible (lowest YM; *p* < 0.05). Tymczewska et al. [26] observed that all samples exhibited greater flexibility compared to pure GL, attributable to the presence of PVA acting as a plasticizer in the mixtures.

Among extract-containing films (FA, FB, and FF), those with lower anthocyanin concentrations, such as FB, showed an increased YM [4]. This behavior is attributable to the ability of phenolic compounds to act as crosslinkers, reinforcing intermolecular interactions within the polymer chains [20]. Non-anthocyanin phenolic compounds possess abundant hydroxyl groups capable of establishing hydrogen bonds with GL-PVA matrix components, thereby increasing YM in FB and FF [37]. FF exhibited the highest YM, likely due to the predominance of non-anthocyanin phenolic compounds in the incorporated extract. A similar trend was observed by Jebel et al. [20] for GL films using eggplant skin extract (ESE), where tensile strength (TS) and YM were significantly increased (*p* < 0.05) at lower ESE concentrations (2–4%).

Conversely, elongation at break (EAB) and tensile strength at break (TB) were highest for the PVA films (*p* < 0.05), a desirable trait for packaging, which must maintain integrity under mechanical stress. PVA contributed more significantly to TB and EAB than GL, a finding consistent with Oyeka et al. [28]. Films formulated with PVA presented the highest TB and EAB values, likely due to PVA’s high crystallinity and polymer network formation, especially used as pristine or in formulations with minimal additive content. However, these mechanical properties are also affected by factors such as hydrolysis degree, molecular weight, plasticizers content, and processing conditions (drying time and temperature).

Similarly, blend films, regardless of blueberry extract addition, presented intermediate EAB values, and the TB values of FC, FB, FA, and FF were statistically equivalent to GL films (*p* < 0.05). In the study by Zeng et al. [10], films incorporating mulberry anthocyanin extracts (MAE) influenced TB and EAB of PVA (10% *w*/*v*), glycerol (1% *v*/*v*), and GL (2% *w*/*v*) films only at higher concentrations (15–45 mg/100 mL); at lower concentrations, no effect was observed. In addition, the decrease in TB and EAB for films FC, FG, FA, FB, and FF compared to FPVA indicates diminished material tenacity, impacting flexibility and tensile resistance [26].

Regarding EAB, it is an important mechanical property for packaging applications, representing the percentage increase in film length before rupture [28]. The FC (GL:PVA 2% wt.; 1:1) exhibited higher EAB values (176%) compared to pure GL films (4% wt.) (7.91%). Dong et al. [38] reported a 76.7% increase in EAB with the addition of 5% PVA to GL films, while Oyeoka et al. [28] also noted that PVA contributed to increasing EAB in blend films. These findings are supported by our data, which show a 96% increase in EAB upon PVA addition.

The addition of blueberry extracts significantly decreased (*p* < 0.05) the EAB of FB, FF, and FA films compared to FC. Tymczewska et al. [26] also observed a slight EAB reduction upon adding black cumin cake extract to a GL:PVA blend (5% wt., 5:3 *v*/*v*) slightly reduced EAB from 137.03% for control films to 125.16%. Although plasticizers such as glycerol generally increase elongation capacity, blueberry extracts may compete for polymer matrix interactions, reducing the plasticizer’s effectiveness and resulting in film that are more prone to rupture during stretching [26].

The TB and YM values for FC were 5.22 MPa and 12.55 MPa, respectively. In contrast, Rashid et al. [25] developed films from PVA (1.25% wt.), GL (3.75% wt.), and GLY (25% wt. relative to polymer mass) that exhibited higher TB (30.20 MPa) and lower YM (0.59 MPa), suggesting improved elasticity and mechanical resistance, suitable for dressing applications. These film properties were similar to those obtained of the FPVA film in this study.

Considering the potential use of the films as pH-sensitive colorimetric indicators [4], reduction in mechanical strength is less critical. Although high tensile strength is advantageous, a balance between structural integrity and biodegradability is essential, as eco-friendly packaging is designed to degrade and minimize environmental impact [28].

High anthocyanin concentrations in films, such as in FA [4], reduced film rigidity due to the predominantly positively charged anthocyanin disrupting PVA-GL interactions and increased molecular mobility, which decreases YM (rigidity) without improving EAB (flexibility) [20,26]. The same effect was observed by Jebel et al. [20] who reported YM reduction in GL films incorporating with 8% eggplant skin extract (rich in anthocyanins).

In summary, the YM of GL- and PVA-based films is influenced by the type of blueberry extract added. FB, FA, and FF films were promising candidates for developing sustainable intelligent films, as they maintained mechanical properties (TB and TK) comparable to the PVA-GL blend and exhibited greater flexibility than FG.

### 3.5. Physical and Chemical Properties

**Figure 6:**
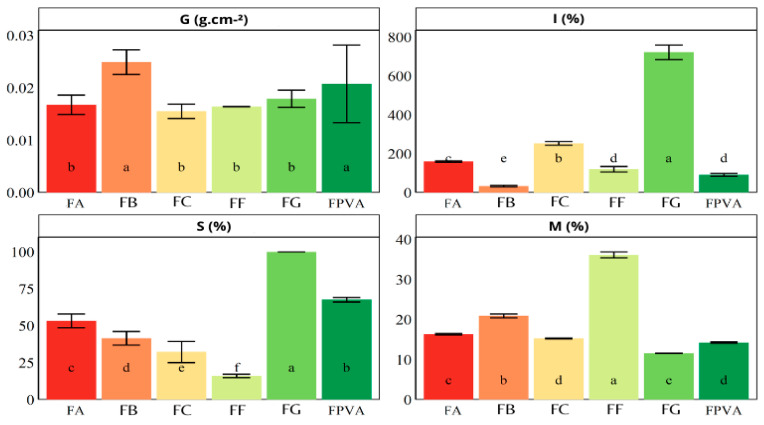
Moisture content (M), solubility (S), grammage (G), and swelling index (I) of control films and films added with blueberry extract. FC (film produced with the PVA and gelatin), FPVA (film produced with the PVA), FG (film produced with the gelatin), FB (film produced with the crude extract), FF (film produced with the phenolic extract), and FA (film produced with the anthocyanin extract). Means followed by the same letter, in the bar, do not differ statistically according to the Scott–Knot test at a significance level of 5%.

The moisture content (M), swelling index (I), solubility (S), and grammage (G) of the control films and of the PVA and GL films added with blueberry extracts are displayed in Figure 6.

According to the results, the moisture content (M) of the films ranged from 11.55% to 36.17%. Phenolic acids and flavonoids typically contain more free hydroxyl groups (-OH) than anthocyanins, which enables the formation of more hydrogen bonds with water in the polymer suspension and enhances water retention within the film matrix. Consequently, the FF film, enriched with phenolic compounds (excluding anthocyanins), exhibited the highest moisture content, whereas the FA film, containing anthocyanin-rich extract, had the lowest moisture content. The FB film, incorporated with blueberry crude extract, presented intermediate moisture content, as the interaction among phenolic acids, flavonoids, and anthocyanins tended to limit water binding more than FF, but less so than FA. In the study by Tymczewska et al. [26], the moisture content of FC (5% wt. PVA and 5% wt. GL; 5:3 *v*/*v*) was 18.09%, which is comparable to the 15.4% found in this study.

Both gelatin and PVA are hydrophilic polymers, meaning that films formed from these polymers have an affinity for water. Their hydrophilicity is attributed to the presence of numerous hydroxyl groups, which confer high polarity and water absorption [28]. The relatively high moisture content observed in pure GL films can be attributed to the abundance of –OH groups. When exposed to water, these groups promote swelling in the PVA and GL films [25]. Moreover, the pure GL film exhibited the highest swelling index (I%) due to electrostatic repulsion between adjacent amine groups in GL, which become positively charged in an aqueous environment and may accelerate water uptake [39].

The FB film had a higher grammage, which may have contributed to its lower swelling index by slowing water penetration and limiting the number of available voids for hydrogen bonds. The presence of phenolic compounds in the blueberry extracts also contributed to a lower swelling index in the FC blend [40], as these compounds bind to free hydroxyl groups in the matrix and in GLY, restricting polymer mobility and hindering water diffusion [41]. Additionally, FA exhibited the highest swelling index, likely due to electrostatic repulsion between positively charged amine groups and in GL, which may increase matrix expansion.

The FF film exhibited the lowest water solubility, attributed to strong hydrogen bonding between non-anthocyanin phenolic compounds and GL and PVA chains, besides GLY, reducing the availability of free hydroxyl groups for interaction with water [20]. In contrast, FB and FA showed higher solubility than FC, as anthocyanins in these formulations have a weaker affinity for the polymer matrix and GLY, form fewer hydrogen bonds, and share a net positive charge with GL, leading to electrostatic repulsion. This repulsion creates more free volume within the polymer matrix, facilitating water entry and enhancing solubility. Since FA contains more anthocyanins than FB, it has the highest solubility.

Overall, the physical and chemical properties of GL and PVA-based films are modulated by the type of blueberry extract incorporated. The PVA-GL formed a film with lower dissolution than FG and FPVA films, making it suitable for food packaging applications. Reduced water solubility is advantageous in such applications, as it helps maintain film integrity when in contact with food exudates, particularly in GL and PVA-based films containing blueberry phenolic extracts. For indicator films used in food packaging, moisture resistance is especially desirable given the high water content of many foods.

### 3.6. Evaluation of the Colourimetric Sensitivity of Indicators in Shrimp

**Figure 7:**
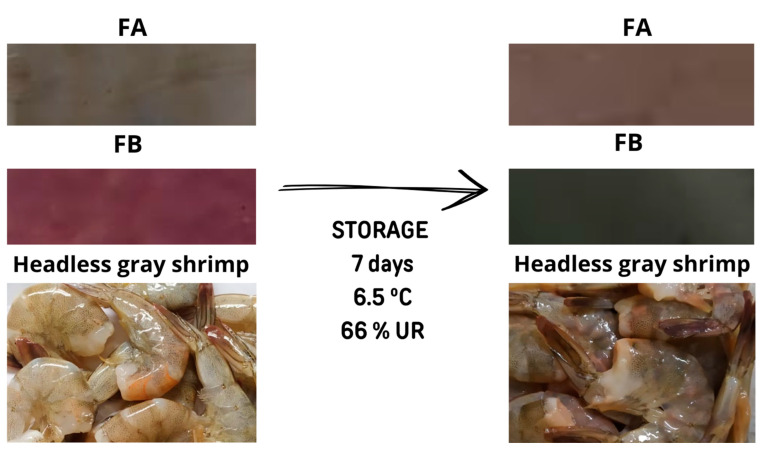
Photographs of the FA and FB indicators and freshness of shrimp, before and after storage under refrigeration (6.5 °C), for 7 days.

The initial appearance of fresh raw shrimp was characterized by a grayish shell with a slightly orange hue, indicative of freshness (Figure 7). As spoilage progressed, degradation was visually observed by darkening of the shrimp surface (ΔE = 5.99 > 3.5) (Figure 7), and by the development of a strong odor, described as ammoniacal, sulfurous (hydrogen sulfide-like), rancid, and putrid. Concurrently, the pH of the shrimp increased from 7.23 ± 0.02 to 7.93 ± 0.02, suggesting ammonia formation due to enzymatic and microbial breakdown of proteins and amino acids, releasing volatile basic nitrogen [42] after 7 days of refrigeration at 6.5 °C. According to the Normative Instruction No. 23 (20 August 2019) from the Brazilian Ministry of Agriculture [43], chilled shrimp must maintain a pH below 7.85 to be considered suitable for consumption. Thus, after 7 days, the shrimp was deemed unfit for consumption.

The colorimetric attributes (L*, a*, b*, C*, h°, and ΔE) of the FA and FB indicator films, as well as the shrimp, after 7 days of storage at 6.5 °C are shown in Table 1. The FA and FB indicators underwent statistically significant color changes (*p* < 0.05), with ΔE values exceeding 3.5, the threshold for perceptible color difference to the human eye. The FB indicator shifted from purple to dark green (ΔE = 20.47), changing hue angle from the fourth to the second quadrant. The FA indicator, with a ΔE of 6.38, changed hue angle from the second to the first quadrant, further confirming that the chromatic changes in this film are visually observable (Figure 7).

The findings of this study are consistent with those reported by [4], who also observed that PVA- and GL-based films incorporated with crude blueberry extract were more sensitive than those incorporated with blueberry anthocyanin extract when exposed to volatile nitrogenous bases, such as those released during shrimp spoilage [42].

Previous studies have elucidated the detection mechanism of indicator films containing anthocyanins for monitoring shrimp freshness. The spoilage of shrimp leads to the generation of volatile nitrogenous bases (ammonia, trimethylamine, and dimethylamine). When these volatile compounds are absorbed by the films, they lead to the production of hydroxyl ions, which induce a color change in the anthocyanins within the polymer matrix [34,44,45].

However, this mechanism does not fully account for the chromatic behavior observed in FA indicators, which are responsive only to acidic vapors. During crustacean spoilage, microbial metabolism also produces volatile acids, such as acetic, propionic, and hydrogen sulfide acids, which contribute to the unpleasant odor of spoiled seafood [42]. As PVA- and GL-based films containing purified blueberry extract are sensitive to acidic vapors, this color change in FA was therefore expected during shrimp degradation [4].

In the study by Teixeira et al. [34], the colorimetric indicators containing açaí anthocyanin extract, plasticized with glycerol and triethylcitrate, changed from pink to gray, and the ∆E values increased in parallel with the loss of freshness of the shrimp, indicating synchrony between the chromatic response and food deterioration, corroborating to the present study. Other pH-sensitive indicators solutions have also been developed to monitor shrimp quality, such as pectin films incorporated with curcumin and sulfur nanoparticles [46] (∆E = 7.1), and films made from chitosan, PVA, zinc oxide nanoparticles, and anthocyanins from purple potato or roselle, which changed from purple to light green during shrimp storage at 4 °C for 8 days [44]. Therefore, the colorimetric indicators developed by incorporating crude and purified blueberry extract into a PVA and GL matrix proved to be an innovative, sustainable, and highly effective solution for quickly, accurately, and practically monitoring the freshness of refrigerated shrimp in real time. This offers an efficient and affordable tool for food quality control, effectively reducing waste.

## 4. Conclusions

The addition of 10% (*v*/*v*) crude, anthocyanin, and phenolic blueberry extracts in FB, FA, and FF films, respectively, did not compromise the thermal stability and mechanical integrity of the films. The thickness (TK) and tensile strength (TB) of the PVA and gelatin films remained consistent with the control formulation (FC). Notably, film flexibility was enhanced compared to the pure gelatin film, alongside a reduction in the swelling index. Among the formulations, FF and FB, both enriched with non-anthocyanin phenolic compounds, exhibited higher moisture content, lower swelling index, reduced water solubility, and increased rigidity compared to FA, which was formulated with purified anthocyanins. FA and FB films demonstrated potential as freshness indicators under controlled laboratory conditions for shrimp. Additionally, FB displayed the most significant and easily perceptible color change in response to shrimp spoilage, outperforming FA in visual sensitivity. This chromatic shift enhances consumer usability, offering a practical and intuitive means to assess food freshness and ensure safety. Fourier transform infrared (FTIR) spectroscopy and cross-sectional microscopy analyses of the films, which would demonstrate the structural and chemical interactions between the polymer matrix (PVA, gelatin, and glycerol) and the blueberry extracts (crude, phenolic, and anthocyanin), were not performed, nor were studies of antimicrobial activity or compound migration. These are limitations of the present study. Future research should evaluate the application of these films to other perishable food matrices, such as fish, poultry, and red meat, which also undergo pH changes resulting in loss of freshness and degradation.

## Figures and Tables

**Table 1:** Mean values and standard deviation of the color attributes L*, a*, b*, C*, h, and ΔE of the FB and FA indicator, and shrimps, before and after storage, for 7 days under refrigeration. The comparison was made in pairs (before and after storage).

Color Coordinates Condition	L*	a*	b*	h°	C*	ΔE
FB before storage	44.64 ± 5.95 ^a^	9.24 ± 1.48 ^a^	−3.12 ± 2.47 ^b^	342.94 ± 11.52 ^a^	9.88 ± 2.12 ^a^	0.00 ± 0.00 ^b^
FB after storage	50.26 ± 6.81 ^a^	−6.51 ± 2.95 ^b^	6.87 ± 1.56 ^a^	131.81 ± 8.73 ^b^	9.54 ± 3.02 ^a^	20.47 ± 2.87 ^a^
FA before storage	64.70 ± 4.51 ^a^	−2.49 ± 0.91 ^b^	7.73 ± 1.69 ^a^	108.52 ± 9.01 ^a^	8.19 ± 1.49 ^a^	0.00 ± 0.00 ^b^
FA after storage	68.98 ± 0.79 ^a^	0.76 ± 0.25 ^a^	8.59 ± 0.97 ^a^	84.78 ± 2.12 ^b^	8.62 ± 0.95 ^a^	6.38 ± 2.59 ^a^
Shrimp before storage	46.48 ± 4.29 ^a^	3.98 ± 3.40 ^a^	3.69 ± 1.47 ^a^	49.64 ± 16.79 ^a^	5.56 ± 3.38 ^a^	0.00 ± 0.00 ^a^
Shrimp after storage	44.66 ± 0.61 ^a^	0.21 ± 0.13 ^a^	2.01 ± 0.65 ^a^	83.65 ± 3.75 ^a^	2.03 ± 0.66 ^a^	5.99 ± 3.53 ^a^

Note: Pairs of means followed by at least the same lowercase letter, in the column, do not differ from each other, at 5% significance, using the t Student test. All measurements were carried out in triplicate. Source: prepared by the authors.

## Data Availability

Data are contained within the article.

## Abbreviations

The following abbreviations are used in this manuscript:

ANOVA	Analysis of variance
EB	Elongation at break
EDS	Energy dispersive spectroscopy
FA film	FPVA:GL incorporated with blueberry anthocyanin extract
FC film	Film produced with GLY, PVA and GL
FB film	FPVA:GL incorporated with crude blueberry extract
FG film	Film produced with GLY and GL
FF film	FPVA:GL incorporated with phenolic blueberry extract
FPVA film	Film produced with GLY and PVA.
G	Grammage
GL	Gelatin
GLY	Glycerol
I	Swelling index
M	Moisture
PVA	Polyvinyl alcohol
RH	Relative humidity
SEM	Scanning electron microscope
S	Solubility
TGA	Thermogravimetric analysis
TK	Thickness
TS	Tensile strength
YM	Young’s modulus
